# Semirational Design of SenC to Enhance Organic Selenium Biosynthesis

**DOI:** 10.1111/1751-7915.70130

**Published:** 2025-03-22

**Authors:** Kailin Shao, Xiaobin Yu, Yan Zhao, Ying Zhang, Xiaobo Liu

**Affiliations:** ^1^ The Key Laboratory of Industrial Biotechnology, Ministry of Education, School of Biotechnology Jiangnan University Wuxi Jiangsu China; ^2^ China Federation Supply & Marketing Cooperation Jinan Fruit Research Institution Jinan China; ^3^ School of Food Science and Engineering, Shandong Agriculture and Engineering University Zibo Shandong China; ^4^ Key Laboratory of Metabolic Engineering and Biosynthesis Technology, Ministry of Industry and Information Technology, Nanjing University of Science and Technology Nanjing Jiangsu China

**Keywords:** biosynthesis, fusion expression, organic selenium, SenB

## Abstract

Organic selenium, a bioavailable form of the essential trace element selenium, holds significant potential for improving human health through dietary supplements and functional foods. However, low bioconversion efficiency has primarily limited the biosynthesis of organic selenium compounds. Here, we focused on enhancing the biosynthesis of organic selenium by optimising the expression and activity of two key enzymes, SenB and SenC, involved in the conversion process. We compared several expression systems, including fusion expression and dual‐promoter approaches, and optimised reaction conditions such as temperature, pH and incubation time. Our results showed that mutations of SenC more than doubled enzyme activity, resulting in a corresponding rise in the intermediate SeP. Notably, the fusion expression of SenB and SenC exhibited the highest conversion rate of organic selenium, achieving over 95% under optimal conditions. Our findings provide a basis for organic selenium production through microbial biotechnology.

## Graphical abstract

Selenium is an essential micronutrient in diverse organisms and crucial to life's health. Here, we demonstrated an enhancement of organic selenium biosynthesis by optimising the expression and activity of the two key enzymes, SenB and SenC, through whole‐cell catalysis.

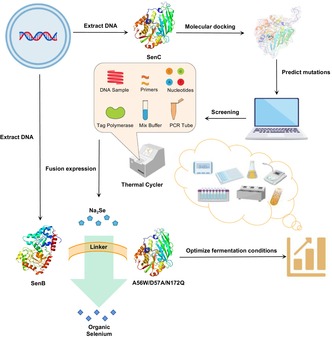

## 1 Introduction

Selenium, a trace element discovered in 1817 by Swedish scientist Jöns Jacob Berzelius (Karaağaç et al. 2022), is essential for human health, playing a critical role in regulating various physiological functions (Roman et al. 2013). Both selenium deficiency and excess intake can significantly impact health. Deficiency is linked to weakened immunity and increased susceptibility to diseases, while excessive selenium intake can lead to toxicity (Santhosh Kumar and Priyadarsini 2014). Selenium is renowned for its antioxidant, immunomodulatory, cardiovascular, antiviral, antiageing and anticancer properties (Kelishadi et al. 2022). Adequate selenium intake supports the immune system (Wang et al. 2023), prevents chronic diseases (Tóth and Csapó 2018) and promotes overall well‐being (Sun et al. 2023).

In recent years, organic selenium has attracted considerable attention due to its superior bioavailability compared to inorganic forms of selenium (Mehdi and Dufrasne 2016). Organic selenium compounds act as powerful antioxidants by scavenging free radicals, protecting cells from oxidative damage, slowing ageing and reducing the risk of chronic diseases (Chen et al. 2021b). In veterinary medicine, organic selenium has been shown to regulate immune‐related gene expression and promote intestinal health (Schrauzer 1976). Notably, organic selenium has demonstrated potential in cancer treatment by enhancing immune response (Arthur et al. 2003), modulating the cell cycle (Köhrle and Gärtner 2009) and aiding in the fight against cancer cells (Schrauzer 1976). Selenium also plays a crucial role in thyroid hormone metabolism (Arthur et al. 1993). In men, selenium improves sperm quality, while in women, it helps regulate the endocrine system (Mistry et al. 2012). Thus, supplementation with organic selenium is essential for maintaining health and preventing disease (Lyons et al. 2007).

Current advances in the biosynthesis of organic selenium have led to significant breakthroughs. Some observations showed that plant extract‐based green synthesis of selenium nanoparticles has gained widespread attention (Pyrzynska and Sentkowska 2022). This led to considerable progress in selenium cyclisation reactions in organic selenium (Chen et al. 2021a). To unveil the mechanisms of biotransformation of selenium, a typical three‐gene cluster was discovered in microorganisms responsible for producing selenoneine (Kayrouz et al. 2022), further expanding the potential for biological selenium utilisation. Other observations showed that the yeast *Yarrowia lipolytica* efficiently converts selenite into stable, biologically active organic selenium nanoparticles, making it a promising platform for biosynthesis (Lashani et al. 2024). Most recently, researchers have transformed a natural sulphur carrier protein‐mediated system into an organic selenide biosynthesis system, enabling the enzymatic synthesis of various organic selenides (Huang et al. 2024).

Importantly, SenB and SenC are pivotal enzymes in the biosynthesis of organic selenium compounds, playing critical roles in the selenium transformation process and the synthesis of selenium‐containing amino acids, such as selenocysteine (Sec) (Eswayah et al. 2016). The enzyme SenB catalyses the selenate reduction to selenite, representing the initial step in selenium metabolism and providing the necessary precursor for subsequent organic selenium compound biosynthesis (Ledesma‐Fernandez et al. 2023). In contrast, SenC facilitates the conversion of selenite to selenium phosphate (SeP) in an ATP‐dependent manner (Kreindl et al. 2024). SeP serves as an essential intermediate for synthesising selenocysteine (Sec). The activity of SenC directly influences the efficiency of Sec biosynthesis, highlighting its critical role in selenium incorporation into proteins (Cain and Krahn 2024). Although these advances open new avenues for drug discovery and functional food development of selenium, the biotransformation of organic selenium compounds is still very limited in yield. This bottleneck essentially blocks the current development of organic selenium compounds in medicine, health care and food industries.

Here, we attempted to optimise the activity of key enzymes SenB and SenC to enhance the biosynthesis of organic selenium. The intermediate product, selenium phosphate (SeP), was determined using various techniques, including HPLC, ICP and ^31^P‐NMR. Site‐directed saturation mutagenesis of SenC was performed via whole‐plasmid PCR, and the mutants were validated for expression. To enhance SeP biosynthesis and the conversion efficiency of organic selenium, four different expression systems, including fusion expression, dual‐promoter, dual‐plasmid and dual‐strain approaches, were employed and compared. Our study provides a basis for improving the biosynthetic efficiency of organic selenium.

## 2 Materials and Methods

### 2.1 Plasmids, Strains and Chemicals

The plasmid pET‐28a(+) was used as the expression vector. *Escherichia coli* BL21 was employed for protein expression, while *E. coli* JM109 was used for gene cloning. The SenC and SenB enzyme genes from *Variovorax paradoxus* DSM 30034 (GenBank accession number AJ811598) were synthesised and purchased from Ningbo Mingzhou Bio Co. Ltd. (Ningbo, China). Molecular biology reagents, including DNA polymerase, were purchased from TaKaRa Bio Inc. (Dalian, China), and Dpn I from BBI Life Sciences Corporation (Shanghai, China). The Bradford Protein Assay Kit was obtained from Beyotime Bio Inc. (Shanghai, China). All other reagents were of analytical grade. Sodium selenide, ATP and UDP sugar were purchased from Aladdin Biochemical Technology Co. Ltd. (Shanghai, China). Primers for gene amplification were synthesised by Sangon Biotech Inc. (Shanghai, China).

### 2.2 Cloning and Expression of Enzymes

Bacterial genomic DNA was extracted, and the target SenB and SenC genes were amplified by PCR. The target genes were then inserted into the plasmid pET‐28a(+) by seamless cloning, with a 6× His tag added at the C‐terminus. Gene cloning was performed using *E. coli* JM109, and gene expression was conducted in *E. coli* BL21 (DE3). Kanamycin (50 mg/L) and IPTG (0.6 mM) were added to the culture medium for selection and induction, respectively. The culture was incubated at 37°C with shaking at 220 rpm until an OD_600_ of 0.6–0.8 was reached. Induction with 0.6 mM IPTG was performed at 20°C for 22 h. Cells were then harvested by centrifugation at 8000 *g* and 4°C for 5 min.

### 2.3 Biosynthesis and Analytical Methods for Intermediates

Whole‐cell synthesis of SeP was carried out in a 1 mL reaction mixture containing 2 mM DTT, 1 mM ATP, 1.5 mM Na_2_Se and 5% (w/v) wet cells (0.5 g, OD_600_ ≈ 20), in 50 mM Tris–HCl buffer. The reaction was incubated at 37°C with shaking at 220 rpm for 6 h. Substrates and intermediates were analysed by HPLC and ICP. ATP consumption during the reaction was used to define enzyme activity.

SeP production was detected using phosphorus ion NMR (^31^P‐NMR) (Klencsár et al. 2018). After 1 h of incubation at room temperature, the reaction was transferred to an NMR tube and analysed immediately under protection from light (Chuai et al. 2021). The results were consistent with previous reports on selenium phosphate synthase (Mirzaeva et al. 2015), confirming the presence of SeP only when all components were present (Banerjee and Koketsu 2017).

ATP in the reaction solution was quantified using an Agilent 1260 HPLC system equipped with an SB‐C18 column (4.6 × 250 mm, 5 μm). The mobile phase consisted of methanol/0.05 mol/L phosphate buffer (1% trifluoroacetic acid) in a 30:70 (v/v) ratio at a flow rate of 0.5 mL/min for 10 min. The absorbance was measured at 259 nm to detect ATP, and compounds were identified by comparing retention times to those of standards (von Papen et al. 2013).

### 2.4 Homology Modelling of SenC and Molecular Docking

**FIGURE 1:**
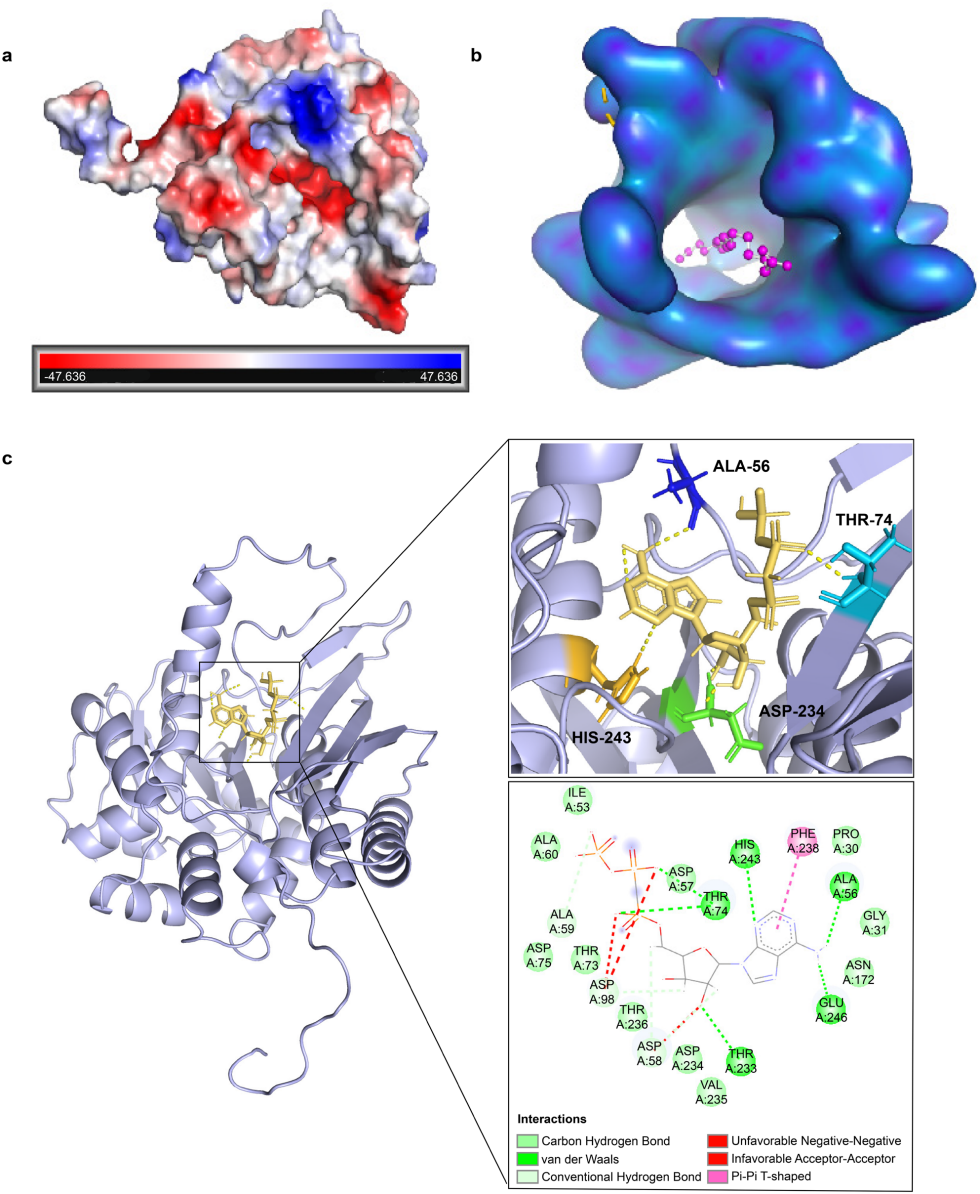
3D structure of SenC and interactions with the ligand. (a) The electrostatic potential surface of the SenC enzyme. The red regions represent negative charges, while the blue areas represent positive charges. (b) The molecular surface of the SenC enzyme shows the ligand‐binding pocket. The ligand is depicted as a magenta‐coloured spherical model. (c) Enlarged view of the binding site between SenC enzyme and ligand (top) and a 2D interaction map (bottom). The interactions between the ligand and key amino acid residues in the binding pocket are shown in detail, including hydrogen bonds (yellow dashed lines), van der Waals forces (green lines), and π–π stacking (pink lines). Key residues such as ALA‐56, THR‐74, HIS‐243, and ASP‐234 are shown as stick models and highlighted in different colours.

The crystal structure of selenophosphate synthase (PDB: 2yyeA) was used as a template for protein homology modelling of SenC using the SWISS‐MODEL server (SWISS‐MODEL, expasy.org) (Waterhouse et al. 2018). The 3D structure of the protein (Figure 1a) was visualised using PyMol (Yuan et al. 2017). The modelling results were evaluated on the SAVES6.0 website (Colovos and Yeates 1993). The ligand AMP 3D structure was obtained from the PubChem database (Kim et al. 2018) (https://pubchem.ncbi.nlm.nih.gov/). Molecular docking of substrate molecules to the protein (Figure 1b) was performed using AutoDock 4.2 software (Eberhardt et al. 2021). The docking results were visualised and analysed using PyMol and Discovery Studio 2019 (Sliwoski et al. 2014) (Figure 1c).

### 2.5 Site‐Directed Saturation Mutagenesis of SenC

Site‐directed mutagenesis of SenC was carried out using whole‐plasmid PCR with pET‐28a(+)‐SenC as the template. Primers for site‐directed saturation mutagenesis are listed in Table S1. The PCR procedure included an initial denaturation at 98°C for 5 min, followed by 35 cycles of 98°C for 10 s, 60°C for 30 s, and 72°C for 30 s, with a final extension at 72°C for 10 min. The PCR products were digested with Dpn I at 37°C for 1 h and then transformed into *E. coli* BL21 (DE3) for expression. Mutants were confirmed by sequencing, and their expression was verified (Figure S1).

### 2.6 Detection of Intermediates

Substrates, intermediates and final products were identified by HPLC and ICP analysis (Klencsár et al. 2018). ATP in the reaction solution was quantified using the same method described above. SeP production was confirmed by phosphorus ion NMR (^31^P‐NMR). After 1 h of incubation at room temperature, the reaction was transferred to an NMR tube and analysed immediately under protection from light. The results were consistent with previous reports on selenium phosphate synthase, confirming the presence of SeP only when all components were present.

### 2.7 Methods for Detection of Biosynthetic End Products

#### 2.7.1 Determination of Total Selenium Content

In this experiment, selenate in the sample was reduced to selenite through acid digestion (Atasoy et al. 2022), followed by further reduction to hydrogen selenide and atomisation. When excited by a selenium lamp (Smrkolj and Stibilj 2004), the fluorescence emitted by selenium atoms was proportional to the selenium content. Selenium concentration was quantified using the standard curve method. The sample (1.00 mL) was subjected to microwave digestion, followed by the addition of hydrochloric acid and potassium ferricyanide solution (Lee et al. 2022), and the final volume was made up to 10 mL (Thavarajah et al. 2011). The selenium content was calculated using the Equation (1) by measuring fluorescence intensity. Results are reported with appropriate significant figures, and the absolute difference between two independent measurements should not exceed 20% of their arithmetic mean.

(1) $$X=\frac{\left(\rho ‐\rho 0\right)\times V}{M\times 1000}$$

*X*, selenium content (mg/kg or mg/L); ρ, selenium concentration in the sample solution (μg/L); ρ0, the selenium concentration in the blank solution (μg/L); *V*, the total volume of the digested solution (mL); *m*, the mass or volume of the sample (g or mL).

#### 2.7.2 Determination of Inorganic Selenium

To extract inorganic selenium, 10.00 mL of the sample was treated using a water bath and ultrasound, followed by centrifugation or filtration. The supernatant was concentrated using hexane extraction to a final volume of 10 mL. The digested solution was mixed with concentrated hydrochloric acid and potassium ferricyanide solution (Tinggi 2003), and the selenium content was measured according to the method described in Equation (2). Results were presented as the arithmetic mean of two independent measurements, rounded to three significant figures, with the absolute difference between the measurements not exceeding 10% of their mean.

(2) $$X=\frac{\left(C‐C0\right)\times V\times 1000\;}{M\times 1000\times 1000}$$

*X*, the selenium content (mg/kg or mg/L); C, the measured concentration of selenium in the sample digest (ng/mL); C0, the measured concentration of selenium in the sample blank digest (ng/mL); *m*, the mass of the sample (g or mL); *V*, the total volume of the digest (mL).

#### 2.7.3 Calculation of Organic Selenium Content

The organic selenium content was calculated by subtracting the inorganic selenium content from the total selenium content. This method assumes that selenium in food is divided into organic and inorganic forms. The organic selenium content is determined by calculating the difference between the total and inorganic selenium concentrations.

### 2.8 Whole‐Cell Catalytic Expression Optimisation

**FIGURE 2:**
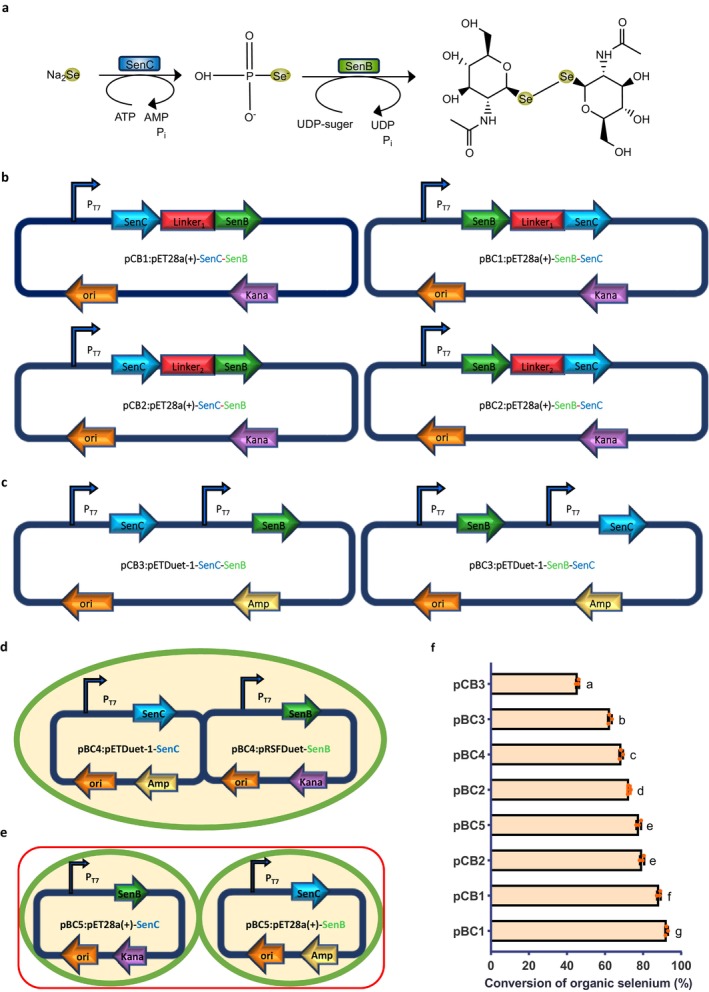
Construction of expression systems for biosynthesis of organic selenium compounds. (a) Biosynthesis pathway of organic selenium compounds. (b) Strains expressing SenC and SenB in a tandem fusion format through various linker peptides and gene combinations. (c) Strains expressing SenB and SenC from dual‐promoter plasmids with different gene combinations. (d) Strains expressing organic selenium compounds through a dual‐plasmid system or coexpression of SenB and SenC in multiple cells. (e) Strains expressing organic selenium compounds via cofermentation of two engineered strains with the same antibiotic resistance. (f) Organic selenium conversion rates of eight different expression system strains. Error bars represent the standard deviation from repeated experiments (*n* = 3). Different letters indicate statistically significant differences (*p* < 0.05, one‐way ANOVA, Tukey's post hoc test).

The experimental reaction was carried out in two modules, relying on selenium phosphate synthase and UDP glycosyltransferase to complete the biosynthetic process. The two enzymes were expressed using different systems, including a fusion expression system, a dual plasmid system and a dual strain system (Kayrouz et al. 2022). The reaction pathway is illustrated in Figure 2a.

#### 2.8.1 Construction of Fusion Protein Expression Strains

Protein fusion can be achieved through various methods, such as direct sequential fusion, linked peptide sequential fusion, embedding fusion, protein‐level fusion and branched fusion (Chen et al. 2013). In this study, sequential fusion with linked peptides was employed. A linker was used as a bridge to fuse the two target enzymes, facilitating the formation of fusion proteins (Shi et al. 2005). The fusion was achieved by introducing the linker between the two proteins in a specific manner. Four plasmid constructs were generated using flexible linkers (GGGGS)_3_ and rigid linkers (PT)_4_P, with pET‐28a(+) as the starting vector. The construction of these plasmids is shown in Figure 2b.

#### 2.8.2 Construction of a Dual Promoter Strain

A dual expression vector was selected to express both enzymes from a single plasmid via seamless cloning. The pETDuet‐1 vector was used for this purpose (Lu et al. 2022). The advantage of the dual promoter plasmid system lies in its ability to express multiple genes simultaneously, enhancing the efficiency of protein expression and simplifying the purification process. Using different promoters for each gene enables the concurrent production of multiple proteins in the same cell, as depicted in Figure 2c.

#### 2.8.3 Construction of a Two‐Plasmid Expression System

The two‐plasmid system offers high efficiency and flexibility (Ng et al. 2000), allowing for the coexpression of multiple genes. This system can also facilitate gene editing and data storage, offering efficient, rapid and nontrace editing (Mamaeva et al. 2023). The pET‐28a(+) and pRSFDuet‐1 plasmids were chosen for the expression of both enzymes. The two‐plasmid system was utilised to study plasmid construction and its impact on enzyme expression, as shown in Figure 2d.

#### 2.8.4 Construction of a Two‐Strain Expression System

Two‐strain coupled fermentation was employed to express the two enzymes in separate bacterial strains. This method offers several advantages, including high selectivity, enhanced catalytic activity and cost‐effectiveness. Additionally, it reduces the need for traditional enzyme‐catalysed cell lysis and protein concentration steps (Chen et al. 2024). In this system, the pET‐28a(+) plasmid was used to express each enzyme in different strains, which were then fermented together using a dual bacterial conjugation system, as illustrated in Figure 2e.

### 2.9 Statistical Analysis

All data processing in this experiment was performed with three independent biological replicates (*n* = 3) to ensure the reliability of the results. The experimental data are presented as the mean ± standard deviation (SD), with error bars representing the standard deviation of these three independent biological replicates, visually indicating the degree of data dispersion. To assess whether there were statistically significant differences between treatment groups, we performed a one‐way analysis of variance (ANOVA). This method is suitable for comparing the means of three or more independent samples. Based on the ANOVA results, if significant differences between groups were identified (typically with a threshold of *p* < 0.05), we further conducted pairwise comparisons using Tukey's HSD post hoc test. The Tukey test controls for the accumulation of Type I errors (false positives) due to multiple comparisons, allowing for a more accurate determination of which groups show true differences. By combining ANOVA with the Tukey test, we can effectively analyse the experimental data and draw reliable statistical conclusions.

## 3 Results and Discussion

### 3.1 Comparison of Performance of Expression Systems

All the expression systems were compared to evaluate their performances in the conversion rate of organic selenium. As shown in Figure 2f, the highest organic selenium conversion rate (92.39%) was achieved using the fused expression system, in which SenB and SenC were coexpressed on a single construct (pET‐28a(+)‐SenB‐SenC). This engineered strain was designated as pBC1. Fusion expression involves linking the genes encoding exogenous proteins with an appropriate linker peptide to form a fusion protein, thereby enhancing the precision and efficiency of expression. Here, the flexible peptide‐based fusion system exhibited the best performance, with the pBC1 strain achieving a conversion rate significantly higher than that of other expression systems (*p* < 0.05). This indicates that the fused expression of SenB and SenC significantly enhances the biosynthesis efficiency of organic selenium. These findings are consistent with previous studies, which have demonstrated that fusion expression can improve the efficiency of multienzyme pathways (Liu et al. 2023). By fusing key enzymes within a metabolic pathway, intermediate product diffusion loss is minimised, and metabolic flux is increased (Bugada et al. 2018). Our results further support this concept, suggesting that the fused expression of SenB and SenC likely enhances organic selenium conversion through a similar mechanism.

Flexible peptide linkers demonstrated superior functionality among various fusion systems due to their ability to provide greater molecular flexibility (Rabeharindranto et al. 2019). This flexibility facilitates better induced‐fit interactions with receptor proteins, enabling the formation of optimal cofactor channels. Such designs bring enzymes into closer spatial proximity, enhancing cofactor transfer and promoting enzymatic reactions within multienzyme complexes (Guo et al. 2024a). Adjusting the length and sequence of linkers minimises interference between domains, allowing precise control over protein folding and activity. The advantage of flexible peptide linkers lies in their ability to optimise the spatial organisation of multienzyme complexes, which is critical for efficient enzymatic reactions. Studies have shown that flexible linkers effectively connect multiple enzymes while maintaining their activities (Chen et al. 2013). For instance, using (GGGGS)_n_ linkers to connect different enzymes has been shown to minimise steric hindrance and preserve catalytic activity.

Our findings are consistent with these observations, suggesting that the (GGGGS)_3_ linker used in this study likely optimised the spatial arrangement of SenB and SenC, thereby enhancing catalytic efficiency. Flexible linkers improve protein folding, stability and activity by maintaining appropriate distances between functional domains, which aligns with our experimental results. Both the pBC1 and pCB1 systems demonstrated higher conversion rates compared to other co‐expression strategies, highlighting the superior performance of these constructs. This supports the notion that flexible linkers enhance protein folding, stability and activity by maintaining optimal distances between functional domains. Experimental results showed that pBC1 exhibited the most favourable expression profile, indicating its potential for further optimisation. These findings will guide the design of subsequent experiments to refine and validate the system's efficiency. In contrast, the pBC2 and pCB2 systems, designed as rigid peptide linker‐based coexpression vectors, did not yield optimal results. While partially effective, rigid peptide‐based fusion systems exhibit adaptability limitations (Guo et al. 2021). They may restrict the conformational flexibility required for optimal enzyme activity, reducing overall efficacy (Chen et al. 2013). For instance, overly short or inflexible linkers can obstruct enzymatic active sites or increase steric hindrance, reducing catalytic efficiency.

The dual‐strain cofermentation system pBC5 followed closely behind, which enhances the yield and quality of metabolic products by leveraging microbial interactions (Yao et al. 2021). The dual‐strain cofermentation system is challenging to operate due to the medium's rapid and extensive consumption of nutrients (Smid and Lacroix 2013). Key factors such as substrate concentration and medium composition are difficult to control, and the system shows moderate performance in this experiment. Therefore, it is not suitable for further experimental work. However, while dual‐plasmid and dual‐promoter systems provide flexibility and control in genetic engineering, they impose significant metabolic burdens on engineered strains, which likely accounts for their lower conversion rates compared with the fused expression system (Selas Castiñeiras et al. 2018). These systems can lead to imbalances in gene expression levels, thereby increasing metabolic stress on host cells (Guo et al. 2024b). In dual‐plasmid pBC4 systems, cells must maintain the replication and expression of two plasmids, which imposes a significant metabolic burden. This results in reduced expression levels of both enzymes, thereby affecting product yield. The dual‐promoter system pBC3 and pCB3 often results in an imbalance in gene expression, where one enzyme is expressed while the other is not. This imbalance imposes excessive metabolic stress on the cells, hindering the completion of the entire biosynthetic process and ultimately leading to the poorest outcomes.

### 3.2 Whole‐Cell Biocatalytic Synthesis of Organic Selenium

**FIGURE 3:**
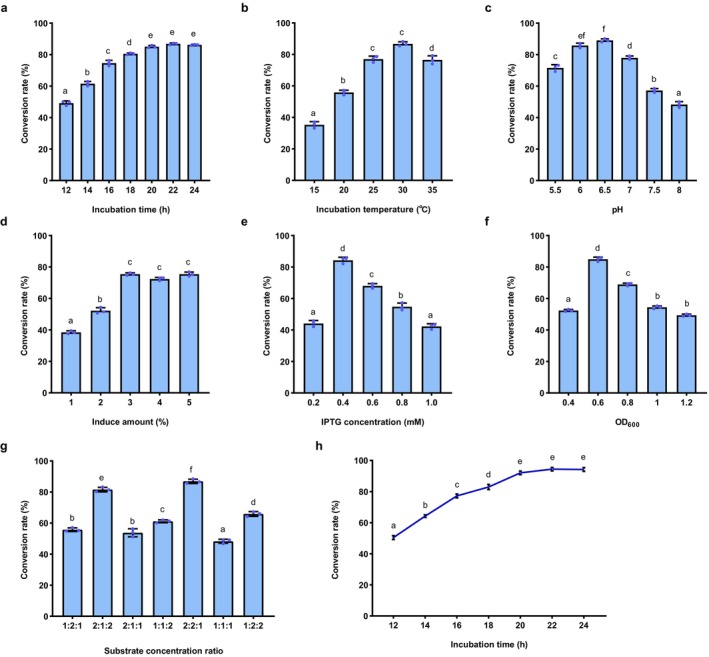
Optimisation of whole‐cell biocatalysis in pBC1 strain. (a) Incubation time. (b) Incubation temperature. (c) Reaction pH. (d) Inoculum size. (e) IPTG concentration. (f) Initial OD_600_ during induction. (g) Effect of substrate concentrations (selenite, ATP, and UDP sugar) on organic selenium conversion rates. (h) Time course of conversion rate under optimal conditions. Time course of enzyme activity under optimal conditions. The data are presented as the mean ± standard deviation from repeated experiments (*n* = 3). Different letters indicate statistically significant differences (*p* < 0.05, one‐way ANOVA, Tukey's post hoc test).

To fully maximise the potential of SenB and SenC for producing organic selenium, we systematically optimised the reaction conditions for strain pBC1. Key parameters were evaluated, such as temperature, pH, incubation time, inoculum size, induction timing, IPTG concentration and substrate ratios. We demonstrated a significant improvement in the conversion rate of organic selenium under the following optimised conditions: an incubation time of 20 or 22 h (Figure 3a), an incubation temperature of 30°C (Figure 3b) and a pH of 6.5 (Figure 3c), which exhibited a bell‐shaped effect on conversion efficiency. Additionally, a 3% inoculum size resulted in the highest conversion rate (Figure 3d). Induction with 0.4 mM IPTG at an OD600 of 0.6 was optimal (Figure 3e,f). For substrate ratios, a combination of selenide, ATP and UDP sugar in a 2:2:1 or 2:1:2 ratio produced the best results (Figure 3g). Based on these conditions, the organic selenium conversion rate increased significantly, reaching up to 94.42% (Figure 3h), a substantial improvement compared to the initial unoptimised conditions (Figure 2f). This optimisation highlights the importance of carefully controlling reaction parameters to maximise biocatalytic efficiency.

The optimal cultivation time was between 20 and 22 h, and the perfect cultivation temperature was 30°C, indicating that these conditions effectively balance enzyme activity and stability. The bell‐shaped pH effect further supports this, suggesting the presence of an optimal range where the activities of both SenB and SenC enzymes are maximised and enzyme stability is highest (Gu 2024). When the temperature is too high or too low, enzyme denaturation or activity reduction may occur, which likely explains the decrease in efficiency beyond the optimal range (German et al. 2011). A 3% inoculum resulted in the highest conversion rate, indicating that this inoculum level provided the optimal cell density, promoting enzyme expression and reaction efficiency. Inadequate inoculum could lead to insufficient enzyme expression, while excessive inoculum could lead to overgrowth of cells and increased resource competition, thus limiting reaction efficiency (Mould et al. 2005). At an OD600 of 0.6, a 0.4 mM IPTG induction concentration was the most effective. IPTG is commonly used in bacterial expression systems to trigger recombinant protein production. This concentration likely represents a balance where sufficient enzyme expression is achieved without overburdening the cellular system, thereby preventing stress‐induced decreases in enzyme activity (Larentis et al. 2014). The selenium compound, ATP, and UDP‐sugar at a 2:2:1 or 2:1:2 ratio produced the best results (Figure 3g). These findings suggest that the substrate ratio directly affects the efficiency of organic selenium conversion. Imbalanced substrate concentrations may create bottlenecks in the reaction, either due to an insufficient supply of one substrate or the excess of another, which subsequently reduces overall efficiency (Shitu et al. 2009).

### 3.3 Optimal Reaction Conditions for SenC

**FIGURE 4:**
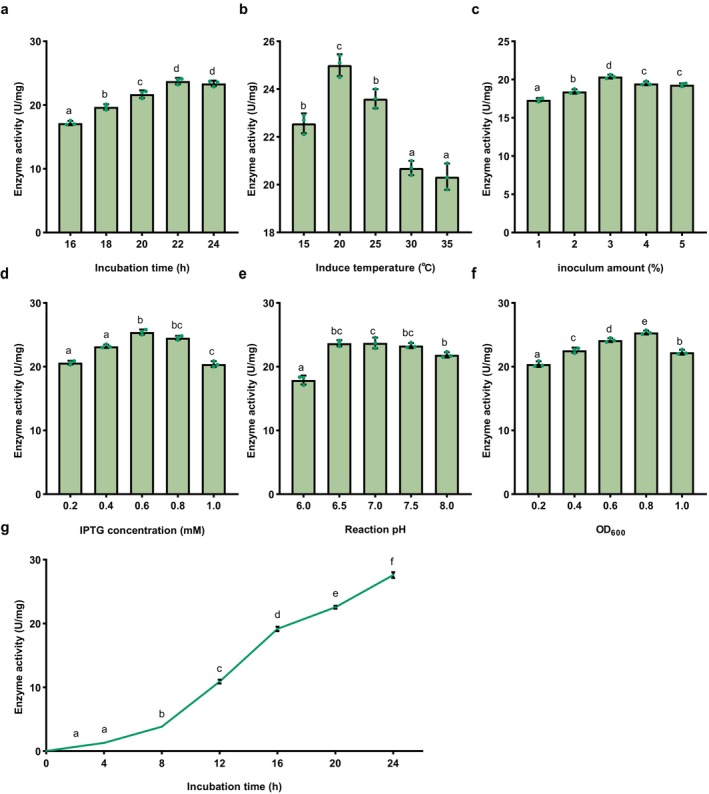
Optimisation of whole‐cell biocatalysis of SenC enzyme. (a) Incubation time. (b) Incubation temperature. (c) Inoculum size. (d) Inducer concentration (IPTG). (e) Reaction pH. (f) Initial OD_600_ during induction. (g) Time course of enzyme activity under optimal conditions. The data are presented as the mean ± standard deviation from repeated experiments (*n* = 3). Different letters indicate statistically significant differences (*p* < 0.05, one‐way ANOVA, Tukey's post hoc test).

Under whole‐cell catalytic conditions, the enzymatic activity of SenC was determined by the hourly ATP consumption rate at a cell concentration of 50 g/L. The optimal conditions for SenC enzymatic activity were identified as follows: an induction duration of 22 h (Figure 4a), an induction temperature of 20°C (Figure 4b), an inoculum volume of 3% (Figure 4c), an IPTG concentration of 0.6 mM (Figure 4d), a reaction system pH of 7.0 (Figure 4e) an IPTG addition at an OD_600_ of 0.8 (Figure 4f). These optimised conditions resulted in the maximisation of SenC enzyme activity; the maximum enzyme activity of 27.6 U/mg was observed within 24 h (Figure 4g).

Enzymatic activity was the highest at 20°C and decreased with increasing temperature. Lower temperatures favour proper protein folding and expression, while higher temperatures can lead to misfolding and inclusion body formation (Kabir and Ju 2023). The highest enzyme activity was observed at an inoculation rate of 3%. Excessive or insufficient inoculation volumes can adversely affect protein yield, with optimal inoculation balancing cell density and metabolic load (Krishna and Nokes 2001). The optimal induction time was 22 h, when protein expression peaked during the logarithmic growth phase before the cells entered the stationary or decline phase. Mis‐timed induction could negatively affect biomass and protein yield (Guan et al. 2021). Induction at OD_600_ = 0.8 produced the highest enzyme activity. Inducing early or late could affect cell growth and protein expression, making synchronisation with optimal cell density crucial (Padhan et al. 2023). An IPTG concentration of 0.6 mM balanced induction efficiency and bacterial viability best. Higher IPTG concentrations may be toxic to host cells, impacting soluble protein expression (Sivashanmugam et al. 2009).

### 3.4 Evolution and Screening of SenC Mutants

**FIGURE 5:**
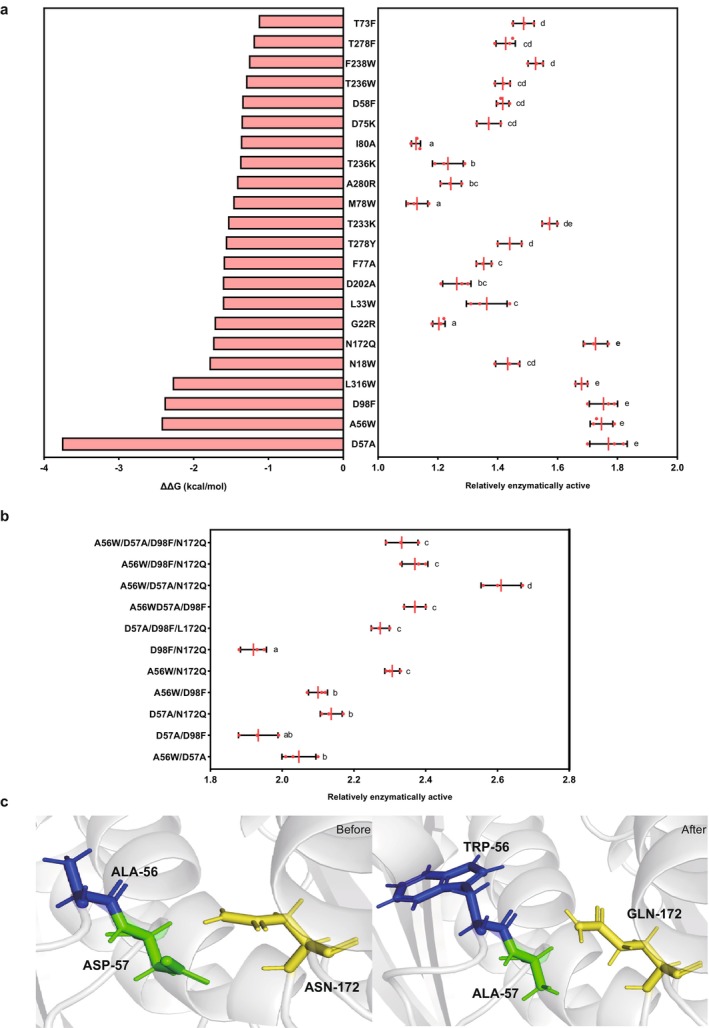
Mutation‐based protein engineering design. (a) Identification of 22 mutation sites through molecular dynamics simulations, along with the relative enzyme activity after introducing each mutation. (b) Relative enzyme activity of combined mutations, highlighting the synergistic or antagonistic effects of specific mutation combinations. (c) Structural morphology of key amino acids (A56, D57 and N172) in the wild‐type protein and the optimal mutant. The visualisation shows the positions and interactions of these residues before and after mutation (Blue: Ala56, Green: Asp57, Yellow: Asn172). The data are presented as the mean ± standard deviation from repeated experiments (*n* = 3). Different letters indicate statistically significant differences (*p* < 0.05, one‐way ANOVA, Tukey's post hoc test).

Using computer simulation software (Guerois et al. 2002), we predicted the key amino acid sites where SenC binds to its substrate. Based on the folding free energy changes of mutation sites predicted by three protein design strategies, mutations with |ΔΔG| < −1 kcal/mol were selected for structural analysis. After eliminating mutation hotspots that introduced adverse interactions and spatial collisions, 22 mutation sites were chosen for experimental verification. The changes in folding free energy for these mutations are shown in Figure 5a.

The amino acid residues D57A, A56W, D98F, L18W, L316W, N172Q, G22R, L33W, D202A, F77A, T278Y, T233K, M78W, A280R, T236K, I80A, D75K, D58F, T236W, F238W, T278F and T73F were selected as the targets, and a mutant library was constructed using the degenerate codon NNK. The relative enzyme activity of the mutants is shown in Figure 5a.

Experimental results indicated that the D57A, A56W, D98F and N172Q mutants exhibited remarkable improvements in activity, with their enzyme activities being 1.77, 1.75, 1.75 and 1.72 times that of the wild‐type enzyme, respectively. These four mutation sites were combined in various forms, yielding 11 combinations. Among these, the mutant D57A/A56W/N172Q demonstrated the highest activity, approximately 2.61 times that of the wild‐type enzyme (Figure 5a). The amino acid morphology before and after mutation for the optimal mutant is shown in Figure 5c.

**FIGURE 6:**
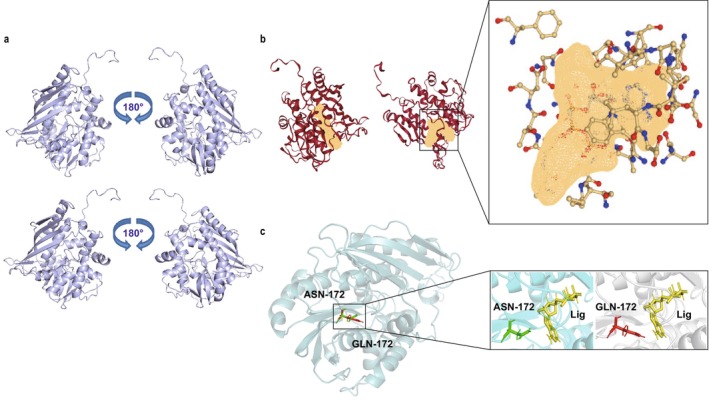
Structural analysis of the wild‐type and mutant SenC. (a) The overall structure of the wild‐type (top) and the A56W/D57A/N172Q mutant (bottom) is presented in two views with a 180° rotation. (b) Schematic representation of the substrate‐binding pocket in the wild‐type (left) and the A56W mutant (right), with an enlarged view of the binding pocket on the right. (c) Comparison of residue N172 (green) in the wild‐type (cyan) and residue Q172 (red) in the A56W/D57A/N172Q mutant (grey), with the ligand shown in yellow.

The three‐dimensional structural models of the wild‐type and mutant variants A56W/D57A/N172Q were obtained using Pymol (Yuan et al. 2017) (Figure 6a). The D98 amino acid site exhibits potential unfavourable interactions that affect the binding affinity between the protein and its substrate. This region is crucial for optimisation in protein engineering design. During the experiment, a certain degree of optimisation was carried out. Single‐point mutations showed promising results, while the effects of combinatorial mutations were less favourable. When A56, D57 and N172 were mutated to smaller alanine residues, the channel entrance widened, reducing steric hindrance and facilitating the entry and release of substances. The volume and width of the binding pocket underwent significant changes, making the binding of the substrate adenosine triphosphate (ATP) to the enzyme tighter, thereby enhancing the enzyme's activity towards this substrate.

ProteinsPlus was also used to analyse the effects of single‐point mutations on the molecular structure (Schöning‐Stierand et al. 2022). The A56W mutation resulted in a 117.44 Å^3^ expansion of the cavity volume, which improved catalytic efficiency. The changes in the size and properties of the amino acids facilitated the movement of substances (Figure 6b) D57, a hydrophilic amino acid, when mutated from hydrophilic aspartate (Asp) to hydrophobic alanine (Ala), led to the breaking of buried hydrogen bonds and loosening of the structure at the entrance, allowing substances to enter more easily. The N172Q mutation‐induced changes in the overall or local spatial conformation of the enzyme, making the three‐dimensional structure of the active site more rational, which favoured the approach and binding of the substrate and more effectively positioned the catalytic groups, thereby lowering the activation energy of the reaction and enhancing catalytic efficiency (Figure 6c).

The combined mutations A56W/D57A/N172Q altered the size, shape and charge distribution of the enzyme's substrate binding pocket, improving its complementarity with the substrate, facilitating the entry and binding of ATP, and promoting the catalytic reaction. When the conformational changes of the binding pocket better matched the substrate structure, the enzyme could more efficiently recognise and bind the substrate, leading to enhanced enzyme activity. Therefore, the three combined mutations significantly expanded the channel size, greatly enhancing catalytic efficiency. The triple mutant displayed a higher yield, which we attribute to the synergistic effect of the three mutations. This also highlights the crucial role of semirational design in mutation optimisation.

## 4 Conclusions

In this study, we engineered strain pBC1, which exhibited the optimal combination of SenC enzymes. Following the optimisation of reaction conditions, the whole‐cell catalytic conversion rate of organic selenium increased from 57.0% to 95.9%. Through molecular dynamics simulations, we identified point mutants (A56W, D57A and N172Q) that outperformed the wild‐type SenC enzyme. Significantly, we developed an outstanding mutant that converted nearly 100 mg/L of inorganic selenium to 95.9 mg/L of organic selenium within 22 h, achieving a conversion rate of approximately 95.9%. This represents the highest conversion rate reported to date. These results demonstrate the success of biosynthetic engineering to enhance the conversion efficiency of organic selenium. The biosynthetic process developed here is highly efficient, environmentally friendly and straightforward. It holds significant potential for expanding the use of organic selenium in synthesising high‐value compounds, paving the way for future applications in various industrial and pharmaceutical fields.

## Author Contributions

**Kailin Shao:** methodology, software, data curation, investigation, writing – original draft, formal analysis. **Xiaobin Yu:** conceptualization, validation, writing – review and editing, supervision. **Yan Zhao:** investigation, writing – review and editing, supervision. **Ying Zhang:** writing – review and editing, data curation. **Xiaobo Liu:** validation, funding acquisition, writing – review and editing, supervision.

## Conflicts of Interest

The authors declare no conflicts of interest.

## Supporting information


Data S1.

## Data Availability

The data that support the findings of this study are available from the corresponding author upon reasonable request.
