# Supplementary material for: Semirational Design of SenC to Enhance Organic Selenium Biosynthesis
Source: Microb Biotechnol. 2025 Mar 22;18(3):e70130. doi: 10.1111/1751-7915.70130 (PMC11929427; doi:10.1111/1751-7915.70130)
Supplement: Supplementary file 1 — Data S1. [file MBT2-18-e70130-s001.docx]

**Supplementary Material for Review**

**Semi-rational Design of SenC to Enhance Organic Selenium Biosynthesis**

Kailin Shao^1^, Xiaobin Yu^1, *^, Yan Zhao^2, *^, Ying Zhang^3^, Xiaobo Liu^4, *^

1. The Key Laboratory of Industrial Biotechnology, Ministry of Education, School of Biotechnology, Jiangnan University, Wuxi, Jiangsu 214122, China;

2. China Federation Supply & Marketing Cooperation, Jinan Fruit Research Institution, Jinan 250200, China;

3. School of Food Science and Engineering, Shandong Agriculture and Engineering University, Zibo, Shandong 255300, China;

4. Key Laboratory of Metabolic Engineering and Biosynthesis Technology, Ministry of Industry and Information Technology, Nanjing University of Science and Technology, 200 Xiaolingwei Street, Nanjing, Jiangsu 210094, China.

^*^Correspondence: Xiaobin Yu (xbyu@jiangnan.edu.cn) or Yan Zhao ([ctcf13011717715@126.com](mailto:ctcf13011717715@126.com)) or Xiaobo Liu ([xbliu@njust.edu.cn](mailto:xbliu@njust.edu.cn))


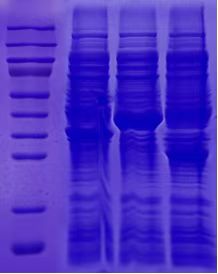


42kDa

33kDa

**Figure S1. The first lane on the left is the marker, the second lane contains SenC enzyme, the third lane contains SenB enzyme, and the fourth lane is the pet28a blank control group.**

|  | **Table S1. All primers used in the plasmid PCR experiments** |
| --- | --- |
| Mutation site | Forward primer & Reverse primer |
| D57A | 5'-cgagacggccgccgacgcggccg-3' |
|  | 5'-cggccgcgtcggcggccgtctcg-3' |
| A56W | 5'-cggcatcgagacgtgggacgacgcggccg-3' |
|  | 5'-cggccgcgtcgtcccacgtctcgatgccg-3' |
| D98F | 5'-caccaacgcgatctccttcgtgtatgcgatgggc-3' |
|  | 5'-gcccatcgcatacacgaaggagatcgcgttggtg-3' |
| L18W | 5'-ccgcctcacctcgtggtcgcacggcggc-3' |
|  | 5'-gccgccgtgcgaccacgaggtgaggcgg-3' |
| L316W | 5'-cagcggcggactgtgggtttcgtgcgcg-3' |
|  | 5'-cgcgcacgaaacccacagtccgccgctg-3' |
| N172Q | 5'-gcgcgtcaagcgccaggccggcgccaagg-3' |
|  | 5'-ccttggcgccggcctggcgcttgacgcgc-3' |
| G22R | 5'-gtcgcacggccgcggctgcgg-3' |
|  | 5'-ccgcagccgcggccgtgcgac-3' |
| L33W | 5'-cgcgcccggcgtgtggtcggaaatcctc-3' |
|  | 5'-gaggatttccgaccacacgccgggcgcg-3' |
| D202A | 5'-agccggccgcggcgagtgcctcc-3' |
|  | 5'-ggaggcactcgccgcggccggct-3' |
| F77A | 5'-gccaccaccgacttcgccatgccgatcgtcga-3' |
|  | 5'-tcgacgatcggcatggcgaagtcggtggtggc-3' |
| T278Y | 5'-gcggccggatgcgccatagacgtgtcccgactg-3' |
|  | 5'-cagtcgggacacgtctatggcgcatccggccgc-3' |
| T233K | 5'-aagcccgtcacgtccttcagcgcatgcacg-3' |
|  | 5'-cgtgcatgcgctgaaggacgtgacgggctt-3' |
| M78W | 5'-atcgtcgacgatcggccagaagaagtcggtggtg-3' |
|  | 5'-caccaccgacttcttctggccgatcgtcgacgat-3' |
| A280R | 5'-gttgcggccggatctgccggtgacgtgt-3' |
|  | 5'-acacgtcaccggcagatccggccgcaac-3' |
| T236K | 5'-gccgaagcccttcacgtcggtcagcg-3' |
|  | 5'-cgctgaccgacgtgaagggcttcggc-3' |
| I80A | 5'-gaacggatcgtcgacggccggcatgaagaagtcg-3' |
|  | 5'-cgacttcttcatgccggccgtcgacgatccgttc-3' |
| D75K | 5'-cgatcggcatgaagaacttggtggtggcgatcagc-3' |
|  | 5'-gctgatcgccaccaccaagttcttcatgccgatcg-3' |
| D58F | 5'-gtagacggccgcgaagtcggccgtctcg-3' |
|  | 5'-cgagacggccgacttcgcggccgtctac-3' |
| T236W | 5'-caagccgaagccccacacgtcggtcagc-3' |
|  | 5'-gctgaccgacgtgtggggcttcggcttg-3' |
| F238W | 5'-cggccaagccccagcccgtcacgtcggtca-3' |
|  | 5'-tgaccgacgtgacgggctggggcttggccg-3' |
| T278F | 5'-gccggatgcgccgaagacgtgtcccgac-3' |
|  | 5'-gtcgggacacgtcttcggcgcatccggc-3' |
| T73F | 5'-catgaagaagtcggtgaaggcgatcagcgcctgc-3' |
|  | 5'-gcaggcgctgatcgccttcaccgacttcttcatg-3' |
